# Supplementary material for: Evaluation of six different tests for Schistosoma haematobium diagnosis in a near-elimination setting: A prospective observational diagnostic accuracy study
Source: PLoS Negl Trop Dis. 2026 Sep 15;20(9):e0014066. doi: 10.1371/journal.pntd.0014066 (PMC13577590; doi:10.1371/journal.pntd.0014066)
Supplement: Supplementary file 1 [file pntd.0014066.s001.pdf]

Table S1: Characteristics of samples identified as positive with urine filtration (UF)-microscopy in a prospective diagnostic accuracy study conducted in Pemba, Tanzania, in 2025.

Characteristics of comparative samples that were identified as positive with a single urine UF-microscopy (52) and with 5-day UF-microscopy (85) among the 262 participants of a prospective diagnostic accuracy study conducted for *S haematobium* in Pemba, Tanzania, in 2025. LIQR: lower interquartile range; UF: urine filtration; UIQR: upper interquartile range.

		**Total** (*n* = 262)	
**Variable**		***N***	**%**
***S. haematobium* infection diagnosed with a single UF-microscopy in same comparative sample**	Positive in same comparative sample	52	19.9
	Mean egg count of positive (range)	169.1 (1-2965)	
	Median egg count of positive (LIQR–UIQR)	51.5 (8-133.5)	
***S. haematobium* infection diagnosed with five UF-microscopy examinations across five days**	Positive in at least one of five samples	85	32.4
	Mean of mean egg counts from five samples (range)	71.6 (0.2-1313)	
	Median of mean egg counts from five samples (LIQR–UIQR)	12.2 (3.6-40.8)	
