# Supplementary material for: Evaluation of six different tests for Schistosoma haematobium diagnosis in a near-elimination setting: A prospective observational diagnostic accuracy study
Source: PLoS Negl Trop Dis. 2026 Sep 15;20(9):e0014066. doi: 10.1371/journal.pntd.0014066 (PMC13577590; doi:10.1371/journal.pntd.0014066)
Supplement: Supplementary file 2 [file pntd.0014066.s002.pdf]

Figure S1: Flowchart of participant and sample selection in a prospective diagnostic accuracy study conducted in Pemba, Tanzania, in 2025.

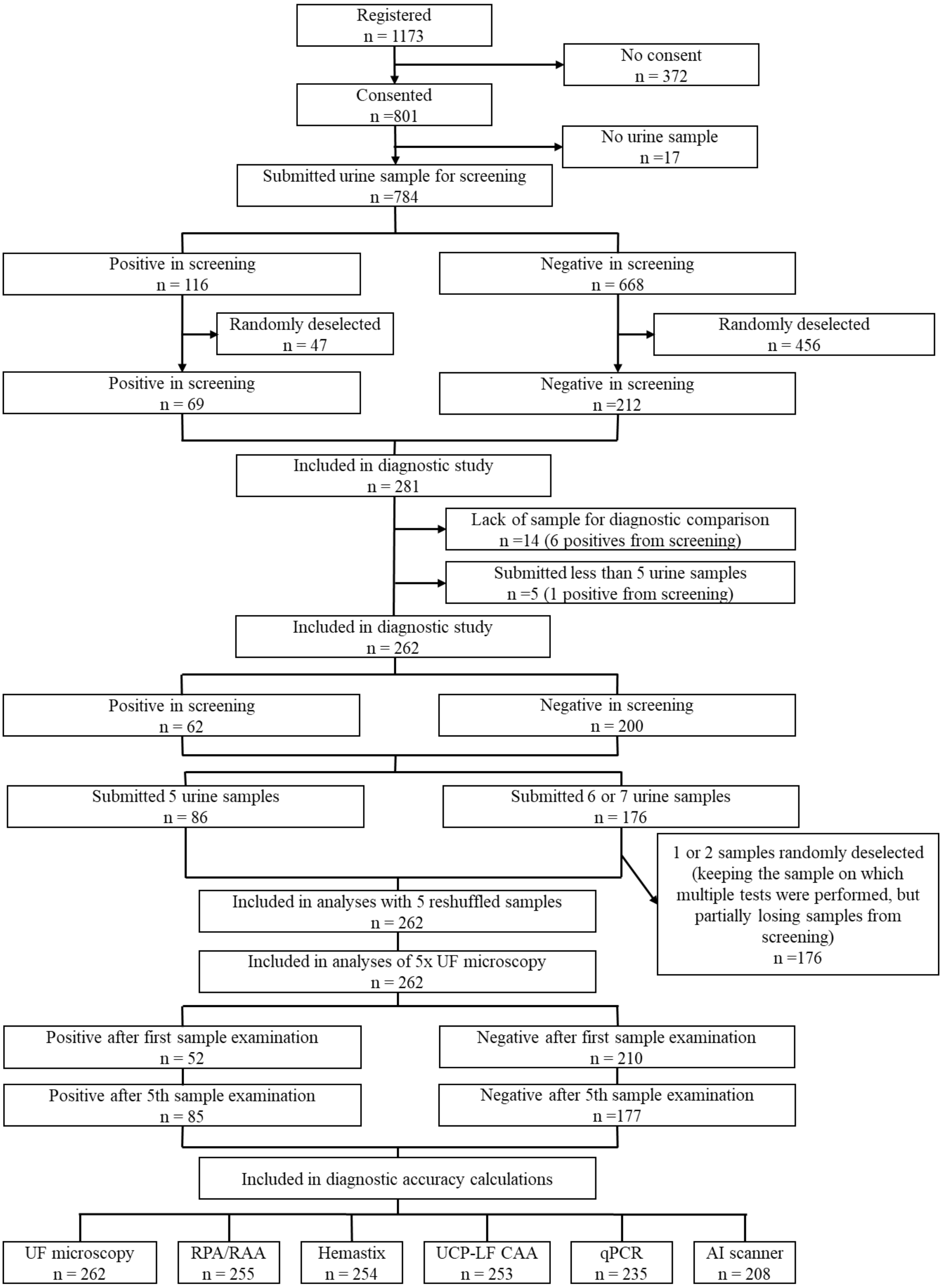

AI-scanner: artificial intelligence-based slide scanner; Hemastix: reagent strips; qPCR: *Schistosoma* ITS-2 real-time PCR; RPA: *S. haematobium* Dra1 recombinase polymerase amplification; UCP-LF CAA: up-converting reporter particle lateral flow circulating anodic antigen assay; UF-microscopy: urine filtration microscopy.
