# Supplementary material for: Evaluation of six different tests for Schistosoma haematobium diagnosis in a near-elimination setting: A prospective observational diagnostic accuracy study
Source: PLoS Negl Trop Dis. 2026 Sep 15;20(9):e0014066. doi: 10.1371/journal.pntd.0014066 (PMC13577590; doi:10.1371/journal.pntd.0014066)
Supplement: Supplementary file 4 [file pntd.0014066.s006.pdf]

Ndum et al. **"**Evaluation of six different tests for *Schistosoma haematobium* diagnosis in a near-elimination setting: a prospective observational diagnostic accuracy study" (Your reference no. PNTD-D-26-00352) **– Point-by-Point response**

Allschwil, 10 August 2026

Dear Dr Rinaldi,

We were delighted to receive your e-mail dated 23 July 2026, which provided valuable feedback from two external reviewers regarding the aforementioned manuscript. In the meantime, we carefully revised our piece in light of the reviewers' comments and suggestions and feel that the overall quality of our manuscript has been further improved.

Below, please find our point-by-point response, clearly indicating how and where in the manuscript changes have been made (line numbers referring to the tracked changed version of the manuscript). To assist you in readily tracking our amendments in the manuscript, we have marked all changes made by using track changes. Additionally, we submit a clean version of the revised manuscript.

We very much hope that this revised version is now suitable for publication in *PLoS Neglected Tropical Diseases*. Hence, we look forward to your further disposition.

Yours sincerely,

Stefanie Knopp

(on behalf of all authors)

****

Dear Dr. Knopp,

Thank you for submitting your manuscript to PLOS Neglected Tropical Diseases. After careful consideration, we feel that it has merit but does not fully meet PLOS Neglected Tropical Diseases's publication criteria as it currently stands. Therefore, we invite you to submit a revised version of the manuscript that addresses the points raised during the review process.

We thank you and the two external reviewers for the appraisal of our work and the overall positive feedback. We revised our manuscript in line with the journal's instructions and addressed the reviewers' comments. Our point-by-point response is given below.

Please submit your revised manuscript by Sep 21 2026 11:59PM. If you will need more time than this to complete your revisions, please reply to this message or contact the journal office at plosntds@plos.org. When you're ready to submit your revision, log on to https://www.editorialmanager.com/pntd/ and select the 'Submissions Needing Revision' folder to locate your manuscript file.

We have submitted our revised manuscript along with a point-by-point response to the reviewers' comments well ahead of the indicated deadline.

Please include the following items when submitting your revised manuscript:

* A letter that responds to each point raised by the editor and reviewer(s). You should upload this letter as a separate file labeled 'Response to Reviewers'. This file does not need to include responses to any formatting updates and technical items listed in the 'Journal Requirements' section below.

We have uploaded a "Response to Reviewers" rebuttal letter, which responds to each point raised by the editors and reviewers to the *PloS Neglected Tropical Diseases* online system.

* A marked-up copy of your manuscript that highlights changes made to the original version. You should upload this as a separate file labeled 'Revised Manuscript with Track Changes'.

We have uploaded a marked-up copy of our manuscript that highlights changes made to the original version using track changes and labelled it "Revised Manuscript with Track Changes".

* An unmarked version of your revised paper without tracked changes. You should upload this as a separate file labeled 'Manuscript'.

We have uploaded an unmarked version of our revised paper where we have accepted all our tracked changes and labelled it "Manuscript".

If you would like to make changes to your financial disclosure, competing interests statement, or data availability statement, please make these updates within the submission form at the time of resubmission. Guidelines for resubmitting your figure files are available below the reviewer comments at the end of this letter.

As requested by the journal, we did change our financial disclosure and competing interests statement and made these updates within the submission form at the time of resubmission.

As the corresponding author, your ORCID iD is verified in the submission system and will appear in the published article. PLOS supports the use of ORCID, and we encourage all coauthors to register for an ORCID iD and use it as well. Please encourage your coauthors to verify their ORCID iD within the submission system before final acceptance, as unverified

ORCID iDs will not appear in the published article. Only the individual author can complete the verification step; PLOS staff cannot verify ORCID iDs on behalf of authors.

Thank you very much for this valuable suggestion. We have encouraged our coauthors to verify their ORCID iD within the submission system before final acceptance.

We look forward to receiving your revised manuscript.

Kind regards,

Gabriel Rinaldi, M.D., Ph.D.
Section Editor
PLOS Neglected Tropical Diseases

Shaden Kamhawi
co-Editor-in-Chief
PLOS Neglected Tropical Diseases
orcid.org/0000-0003-4304-636XX

Paul Brindley
co-Editor-in-Chief
PLOS Neglected Tropical Diseases
orcid.org/0000-0003-1765-0002

****

Journal Requirements:

If the reviewer comments include a recommendation to cite specific previously published works, please review and evaluate these publications to determine whether they are relevant and should be cited. There is no requirement to cite these works unless the editor has indicated otherwise.

The reviewers did not include any recommendations to cite specific previously published works.

1) Please amend your detailed Financial Disclosure statement. This is published with the article. It must therefore be completed in full sentences and contain the exact wording you wish to be published.

Our Financial Disclosure statement reads as follows: “Funding for the study has been obtained from the Swiss National Science Foundation (SNSF; Bern, Switzerland) via a PRIMA grant (PR00P3_179753) to SK and by the Leading House Africa via a Consolidation Grant to SK. A grant of the Prof. Dr. P.C. Flu Foundation (8244-30453) in the Netherlands to LvL supported

part of the work dedicated to the qPCR and UCP-LF CAA test. SK received her salary as part of the PRIMA grant (PR00P3_179753) awarded by the SNSF. NCN received part of her salary from a personal stipend from the Swiss Government Excellence Scholarships (ESKAS) programme. The funders had no role in study design, data collection and analysis, decision to publish, or preparation of the manuscript." (**see revised manuscript, lines 593-600 and online submission system**).

i) State what role the funders took in the study. If the funders had no role in your study, please state: "The funders had no role in study design, data collection and analysis, decision to publish, or preparation of the manuscript."

We added this sentence to the funding statement: "The funders had no role in study design, data collection and analysis, decision to publish, or preparation of the manuscript." (**see revised manuscript, lines 599-600 and online submission system**).

ii) If any authors received a salary from any of your funders, please state which authors and which funders.

If you did not receive any funding for this study, please simply state: u201cThe authors received no specific funding for this work.u201d.

We indicated the following: "SK received her salary as part of the PRIMA grant (PR00P3_179753) awarded by the SNSF. NCN received part of her salary from a personal stipend from the Swiss Government Excellence Scholarships (ESKAS) programme." (**see revised manuscript, lines 597-599 and online submission system**).

2)Please send a completed 'Competing Interests' statement, including any COIs declared by your co-authors. If you have no competing interests to declare, please state "The authors have declared that no competing interests exist". Otherwise please declare all competing interests beginning with the statement "I have read the journal's policy and the authors of this manuscript have the following competing interests:"

We have completed our Competing Interests statement as follows: "We have read the journal's policy and the authors of this manuscript have the following competing interests: Peter Ward and John Bergelin are employees of Enaiblers AB (Uppsala, Sweden) and hold shares or share options in the company. Enaiblers AB is a social enterprise that develops and markets affordable integrated diagnostic tools for NTDs, including the AI-scanner evaluated in this study. All other authors have declared that no competing interests exist." (**see revised manuscript, lines 602-608 and online submission system**).

REVIEWER REPORTS

Reviewer #1:

No Response

We thank Reviewer #1 for evaluating our work. We take it as a positive sign that no specific comments to the authors were provided.

Reviewer #2:

Line 112 – picky, but presumably the parents or guardians were told about the children's status and offer on medication?

We realize that the information provided in our ethical section was too short for accurate understanding of the procedures. To enhance clarity, we have amended the section as follows: "Before the onset of the study, information meetings were held with principals, teachers and parent delegations of the two study schools in Pemba Island, United Republic of Tanzania, to explain the study purpose and procedures and to discuss any resulting questions or concerns. The study was also explained in lay terms to students from grades 3, 4, 5 or 6 who were eligible to participate. They received an information sheet in Kiswahili (local language) and an informed consent form for their parents/legal guardians to sign if they agreed to the participation of their child in the study. In addition to their parents' written consent, children aged 12-17 years were invited to submit an assent form signed by themselves. At the end of the study, all participants who tested *S. haematobium*-positive were treated with praziquantel (40 mg/kg)." (**see revised manuscript, lines 111-119**).

Of note, the ethical board from the Zanzibar Health Research Institute (ZAHRI) explicitly requested that only children who tested *S. haematobium*-positive in this diagnostic study were treated with praziquantel. Blanked treatment of all students without prior diagnosis is conducted by the NTD programme of the Zanzibar Ministry of Health annually in all primary schools.

Line 119 – 60 positive and 190 neg, line 134 - 70 positive, 180 neg
Ah, I see in next paragraph, perhaps this needs to be written to be clear that the 60 is presuming a level of false negatives which will be revealed upon additional multi-day testing.

We thank Reviewer #2 for this suggestion and revised as follows: "Hence, to identify 60 *S. haematobium*-positive students in the initial cross-sectional screening based on the examination of one urine sample per participant with UF-microscopy (where a level of false-negatives is presumed) and to obtain the required sample size of 70 *S. haematobium*-positive participants after examination of five urine samples per participant with UF-microscopy (where some initially false-negatives will be revealed as positive), we estimated that we needed to screen 1,200 students from two schools (600 students per school)." (**see revised manuscript, lines 144-150**).

Figure 1 – was there a prioritization schedule for the different diagnostics? Just noting variations in how many tests were performed for each.

We thank Reviewer #2 for this question. Yes, there was a prioritization schedule for the diagnostics aligned with the amount of urine available and to be used for the test., which we now indicated in Table 1 (**revised manuscript, lines 185-187 and Table 1**). However, there was also a restriction in the number of samples that could be examined with certain methods due to costs (i.e. qPCR) and some loss of samples during the processes indicated in the flowcharts in Fig 1 and S1 Fig. The numbers that are shown in the bottom line of the flowcharts in Fig 1 and S1 Fig indicate the final number included in the statistical analyses of this manuscript (**revised manuscript, Fig 1 and S1 Fig**).

**Priority**	**Test**	**Volume**	
Priority 1	UF-microscopy	10	ml
Priority 2	UCP-LF CAA	4	ml
Priority 3	qPCR	10	ml
Priority 4	RPA	10	ml
Priority 5	AI scanner	10	ml
Priority 6	Hemastix	1	ml

Table 1. Prioritization schedule for the six diagnostic methods applied in a prospective diagnostic accuracy study for *S. haematobium* in Pemba, Tanzania, in 2025.

For testing sens, I wonder if it is worth also doing a combination of tests as a comparator? Particularly as you mention the AI scanner having operator verified eggs. In fact, perhaps you could do just the AI as a second sens and spec method which would better reveal this issue? May clear up the qPCR results as well. Although the latent class analysis also helps.

We thank Reviewer #2 for this point. Although the idea of a verified positive subset is interesting and has certain advantages, it would challenge comparisons with the other diagnostic tests because it would favour the accuracy of the AI scanner. Hence, we decided to keep the presentation of results as in our originally submitted version, refrain from the use of an additional combination of tests as a comparator.

Figure resubmission:

While revising your submission, we strongly recommend that you use PLOS's NAAS tool (https://ngplosjournals.pagemajik.ai/artanalysis) to test your figure files. NAAS can convert your figure files to the TIFF file type and meet basic requirements (such as print size, resolution), or provide you with a report on issues that do not meet our requirements and that NAAS cannot fix.

After uploading your figures to PLOS's NAAS tool - https://ngplosjournals.pagemajik.ai/artanalysis, NAAS will process the files provided and display the results in the "Uploaded Files" section of the page as the processing is complete. If the uploaded figures meet our requirements (or NAAS is able to fix the files to meet our requirements), the figure will be marked as "fixed" above. If NAAS is unable to fix the files, a red "failed" label will appear above. When NAAS has confirmed that the figure files meet our requirements, please download the file via the download option, and include these NAAS processed figure files when submitting your revised manuscript.

We used the PLOS's NAAS tool (https://ngplosjournals.pagemajik.ai/artanalysis) to test our figure files as recommended. All our figures of the revised manuscript passed the check and we submitted these NAAS processed figure files together with our revised manuscript.

Reproducibility:

To enhance the reproducibility of your results, we recommend that authors of applicable studies deposit laboratory protocols in protocols.io, where a protocol can be assigned its own identifier (DOI) such that it can be cited independently in the future. Additionally, PLOS ONE offers an option to publish peer-reviewed clinical study protocols. Read more information on sharing protocols at https://plos.org/protocols?utm_medium=editorial-email&utm_source=authorletters&utm_campaign=protocols

Thank you very much for this recommendation.
